# Prognostic value of Ocular Trauma Score and pediatric Penetrating Ocular Trauma Score in predicting the visual prognosis following ocular injury

**DOI:** 10.22336/rjo.2022.29

**Published:** 2022

**Authors:** Aparajita Chaudhary, Rupanshi Singh, Satya Prakash Singh

**Affiliations:** * Department of Ophthalmology, Motilal Nehru Medical College, Prayagraj, U.P., India

**Keywords:** ocular, trauma, OTS, POTS, prognosis

## Abstract

**Aim:** To assess the role of Ocular Trauma Score (OTS) and Pediatric Penetrating Ocular Trauma Score (POTS) in predicting visual prognosis following ocular injury.

**Methods:** 120 patients, aged 3 to 70 years, with ocular injury, presenting between August 2020 and 2021, who fulfilled the inclusion criteria, were classified using the Birmingham Eye Trauma Terminology System (BETTS). Data regarding age, sex, injury type, location, initial visual acuity, and treatment were recorded. Patients were evaluated using OTS and POTS, wherever applicable, to predict visual prognosis. Follow-up was done for 6 months post-treatment to compare the predicted and actual visual outcome.

**Results:** 120 patients (96 males and 24 females) were included in the study. The mean age was 17.2 ± 14.7 years (range 3 to 70 years). 91.6% patients included were under the age of 40 years. Blunt trauma (n=62) was slightly more common. 80 patients (66.67%) reported to the hospital after 48 hours of injury. 34 patients (28.33%) had traumatic cataract alone and IOL implantation was done as a primary procedure. In 20 patients (16.67%), lens aspiration was done primarily, with secondary IOL implantation. Six months after the treatment, the BCVA in the open globe injury patients was 20/ 200 or better in 36 patients (75%) and 20/ 40 or better in 18 patients (37.5%). Amongst the closed globe injuries, 48 patients (66.67%), had a BCVA 20/ 200 or better, while 32 (44.44%) had a BCVA of 20/ 40 or better.

**Conclusion:** OTS is a reliable predictor of final visual outcome, both in blunt and penetrating injuries. The POTS shows gross underestimation of final visual outcome.

**Abbreviations:** BETTS = Birmingham Eye Trauma Terminology System, POTS = Pediatric Penetrating Ocular Trauma Score, OTS = Ocular Trauma Score, OGI = Open Globe Injury, HM = Hand Movement, NLP = No Light Perception, LP = Light Perception, CGI = Closed Globe Injury, VA = Visual Acuity, BCVA = Best Corrected Visual Acuity, IAP = Indian Academy of Pediatrics, IOL = Intraocular Lens, IGATES = International Globe and Adnexal Trauma Epidemiology Study, IOP = Intraocular Pressure, CART = Classification and Regression Tree, USA = United States of America

## Introduction

Worldwide, ocular trauma is an important cause of eye morbidity and is a leading cause of non-congenital, monocular blindness, especially in children. In addition to visual impairment, eye injury is known to cause significant morbidity, in terms of pain, psychosocial stress, and economic burden [**1**]. Not only the trauma victim, but also the family must bear the psychological burden of uncertainty in such times. Moreover, having a reasonably accurate functional prognosis is crucial when the choice between various management decisions must be made, a process in which the patient and the family also participate [**2**].

Ocular trauma includes a wide range of pathologies, ranging from corneo-scleral tears to cataract and posterior segment abnormalities, each with different management protocols and follow-up, and each with a different sequela. Long term, dedicated post-operative evaluation by the ophthalmologist, and patient awareness make an integral part of ocular rehabilitation and patient recovery.

In 2002, a systematic classification, the Birmingham Eye Trauma Terminology System (BETTS) was developed, which classifies the ocular injuries into Open Globe Injury (OGI) and Close Globe Injury (CGI) and defines them and their subtypes [**3**]. Thereafter, based on the standard terminology, a system was framed to help predict the visual acuity (VA) in ocular trauma post treatment. Thus, the Ocular Trauma Score (OTS) was developed. It uses a fixed set of variables, readily determined at the time of initial evaluation of the patient, along with basic mathematics, expected to give the ophthalmologist a 77% chance to predict the final visual outcome ± one visual category, shortly after the eye injury [**4**]. An early estimate of the future prognosis helps in the appropriate counselling of the patient and contributes to making decisions regarding the management.

Eye trauma in children differs significantly from that in adults. It is difficult to obtain sufficient information from children regarding the trauma because of pain and restlessness, and they may not be aware of a reduction in their VA [**1**]. The ophthalmological examination in children is also difficult, compared to adults. Children develop more extensive post operative inflammation, scarring, and proliferative vitreoretinopathy than adults. The visual development continues until the age of 9-10 years, and despite successful trauma treatment, VA may not improve because of amblyopia. In addition, the same trauma in the eye of a child <5 years of age and in a patient of 15 years old does not affect the eye in the same manner [**1**]. Therefore, these factors need to be incorporated while predicting the visual outcome in children. Hence, the Pediatric Penetrating Ocular Trauma Score (POTS) was developed. 

POTS awards fewer points to initial VA and takes into consideration the age of the patient, the wound location and associated pathologies that may result in amblyopia in children. Thus, it is a more elaborate measure of the future functional prognosis of the eye in children with penetrating eye injuries. 

In the past, various models have been used to predict the prognostic visual outcome in trauma patients. These include the OTS, POTS, and the Classification and Regression Tree (CART). However, most of these studies have been a retrospective evaluation of previously recorded patients. The few prospective studies that have been conducted had a small sample size and short follow-up, insufficient to produce reliable results. 

In this study, we have prospectively evaluated ocular trauma patients, of all age groups and pathologies and used the OTS and POTS to predict their future visual outcome. 

## Methods

The study was carried out on a total of 120 patients who attended the ophthalmic clinic of the Department of Ophthalmology, Regional Institute of Ophthalmology (M.D. Eye Hospital, Dr. Katju Road, Nakhas Kona, Prayagraj) from August 2020 to August 2021, who met the inclusion criteria and were found eligible to be included in the study.The approval for this study was obtained from the Ethical Committee of Motilal Nehru Medical College, Prayagraj. A written consent was obtained from all participants before being included in the study.

The inclusion criteria were the following: 

1. Patients of all ages and genders were included in the study.

2. Patients exhibiting clinical signs of damage to any ocular structure following trauma to the eye, including cornea, anterior chamber, or posterior chamber.

3. All the cases had to manifest with diminution of vision following ocular trauma.

4. All the patients signed and dated consents for any procedures undertaken to restore vision. 

5. All the patients who complied with the study procedures and were available during the study.

Exclusion criteria:

1. Any patients suffering no diminution of vision following ocular trauma.

2. Any patients with damage to the integrity of the lid or adnexa but no damage to cornea, anterior chamber, or posterior chamber.

3. Patients who could not participate in the treatment or be monitored frequently according to the study protocol.

4. Patients presenting to the center after the initial treatment elsewhere.

5. Patients with other ocular diseases affecting the visual function.

6. Patients with previous intraocular surgeries.

7. Patients with No Perception of Light.

All the patients underwent a detailed medical history regarding the cause and conditions of the injury and subsequent classification of the injury according to the BETTS. The patients were examined for anterior and posterior segment abnormalities. They underwent Slit lamp Examination, Pupillary reflex test, Indirect ophthalmoscopy with 20D lens, Ultrasound B-scan, and Intraocular Pressure (IOP), wherever necessary.

The Ocular Trauma Score [**2**] for the patients was calculated by assigning raw scores to individual patient characteristics, as mentioned below.

**Table 1:** OTS raw score

	Variables	Raw Points
A.	Presenting Vision	
	20/ 40 or better	100
	20/ 200 to 20/ 50	90
	1/ 200 to 19/ 200	80
	Light Perception (PL)/ Hand Movement (HM)	70
	No Light Perception (NLP)	60
B.	Rupture	-23
C.	Endophthalmitis	-17
D.	Perforating Injury	-14
E.	Retinal Detachment	-11
F.	Afferent Pupillary Defect	-10

For patients up to 18 years of age with Open Globe Injuries, Pediatric Penetrating Ocular Trauma Score [**1**] will be calculated using the following variables:

**Table 2:** POTS raw score

	Variables	Raw Points
A.	Initial Visual Acuity	
	20/ 40 or better	50
	20/ 200 to 20/ 50	40
	1/ 200 to 19/ 200	30
	Light Perception/ Hand Movement	20
	No Light Perception	10
B.	Age of the patient (years)	
	0 to 5	10
	6 to 10	15
	11 to 15	25
C.	Wound Location	
	Zone 1	25
	Zone 2	15
	Zone 3	10
D.	Concomitant Eye Conditions	
	Iris Prolapse	-5
	Hyphaema	-5
	Organic/ Unclean Injury	-5
	Delay of Surgery (≥ 48 hours)	-5
	Traumatic Cataract	-10
	Vitreous Haemorrhage	-20
	Retinal Detachment	-20
	Endophthalmitis	-30

The sum of all raw points provides the Raw Score that helps to classify the patients in OTS and POTS categories and hence predict their visual outcome according to the categories they fall under. The expected visual outcome in different categories is mentioned below [**3**]. 

**Table 3:** Probability table

Sum of Raw Points	OTS/ POTS category	No Light Perception (NLP)	Light Perception/ Hand Movement	1/ 200 to 19/ 200	20/ 200 to 20/ 50	20/ 40 or better
0 to 44	1	73%	17%	7%	2%	1%
45 to 65	2	26%	28%	18%	13%	15%
66 to 80	3	2%	11%	15%	28%	44%
81 to 91	4	1%	2%	2%	21%	74%
92 to 100	5	0%	1%	2%	5%	92%

Follow up was performed to evaluate the visual acuity post treatment (medical or surgical) at 1 week, 2-week, 1 month, 3 months and 6 months. The Best Corrected Visual Acuity (BCVA) was evaluated 6 months after treatment.

At every follow-up visit, patients were examined for:

1. Slit lamp examination.

2. IOP by Schiotz Tonometer.

3. Fundus examination by indirect ophthalmoscope.

4. Refraction under full cycloplegia.

5. BCVA.

6. Post-operative amblyopia treatment by occlusion therapy, if needed.

This predicted the final visual acuity, which was then compared with the patient’s best corrected visual acuity, 6 months after treatment, medical or surgical, to assess the prognostic value of OTS and POTS.

## Results

120 eyes of 120 patients aged 3 to 70 years old underwent treatment for blunt and penetrating ocular trauma ranging from corneal and scleral tear repair to cataract surgery, vitrectomy, and retinal detachment in a one-year period. Out of these 120 patients, 96 were males and 24 were females.

Out of 96 males, 48 suffered blunt ocular trauma, while 48 suffered penetrating ocular trauma. The latter included patients <18 years of age and were evaluated using POTS, while 5 patients >18 years of age were evaluated using OTS. All the patients with blunt ocular injury were evaluated using OTS. 

Out of the 24 female patients, 14 suffered blunt ocular trauma and were evaluated using OTS and 10 suffering penetrating ocular trauma, all of whom were <18 years old, were evaluated using POTS. This is because the Indian Academy of Pediatrics (IAP) recognizes individuals <18 years of age under pediatric category. 

All the patients were thus divided into two groups according to the score used for the initial evaluation of the patient to predict the future visual prognosis:

- Group 1: OTS group that included 72 patients; 

- Group 2: POTS group that included 48 patients.

All the patients received the primary treatment in our center and were not treated elsewhere. Of the injuries, 80 patients (66.67%) reported to the hospital after 48 hours of injury, while 40 (33.33%) reported within the first 24 hours of trauma. 40 patients (33.33%) presented to our side more than 3 months after trauma. Neither the cause nor the place of injury was significantly associated with the final visual acuity. 

**Fig. 1:**
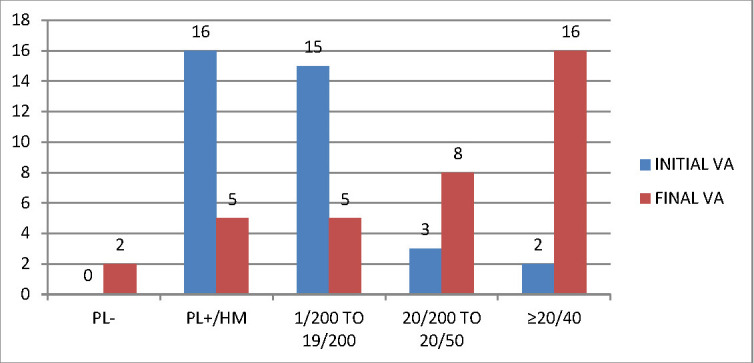
Comparison of final BCVA in OTS subjects. Significant improvement is observed, especially in groups 2 and 3

**Fig. 2:**
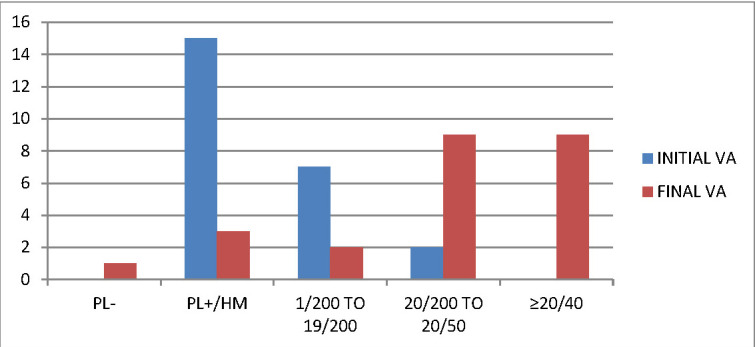
Comparison of final BCVA in POTS subjects. Significantly improved visual outcome is observed, especially in group 2

**Fig. 1** and **2** compare the presenting visual acuity of our patients in both OTS and POTS groups with the final BCVA achieved after treatment. However, we did not include patients with initial VA of NLP in our study. 34 patients (28.33%) had traumatic cataract alone and Intraocular lens (IOL) implantation was performed as a primary procedure. 20 patients (16.67%) presented with corneoscleral tear along with cataract and the lens aspiration was done primarily, but IOL implantation in these patients was done as a secondary procedure, once the inflammation subsided, at least 2 weeks after the primary surgery. IOL implantation was performed in 78 patients (65%) in total. Six months after the surgery, the BCVA in the open globe injury patients was 20/ 200 or better in 36 patients (75%) and 20/40 or better in 18 patients (37.5%). Amongst the closed globe injuries, 48 patients (66.67%) had a BCVA 20/ 200 or better, while 32 (44.44%) had a BCVA of 20/ 40 or better. The follow-up was performed for 6 months. 

**Fig. 3:**
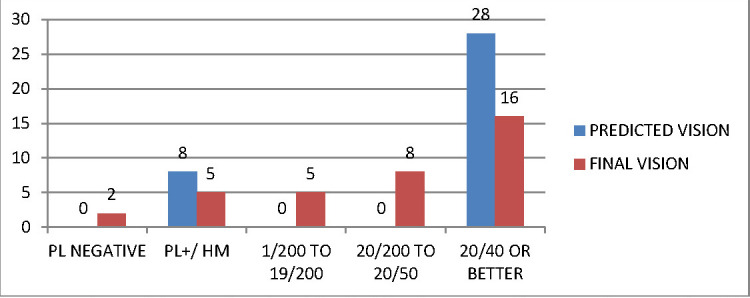
Comparison of the predicted and final vision in OTS subjects. Both categories follow a similar pattern, showing similarities in the predicted and achieved visual outcome

**Fig. 4:**
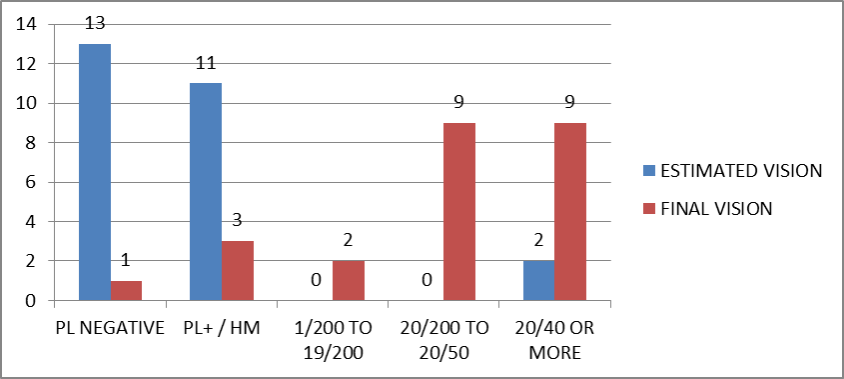
Comparison of the predicted and final vision among POTS subjects. The graph clearly highlights the gross underestimation of visual outcome in children

**Fig. 3** and **4** compare the predicted and Final BCVA according to OTS and POTS predictions in patients.

**Fig. 5:**
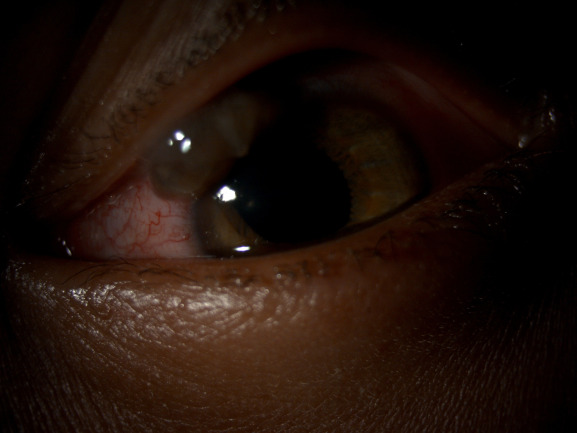
Pre operative photograph of a 3-year-old male with limbal tear caused due to knife injury. The patient presented and was operated within 24 hours of injury

**Fig. 6:**
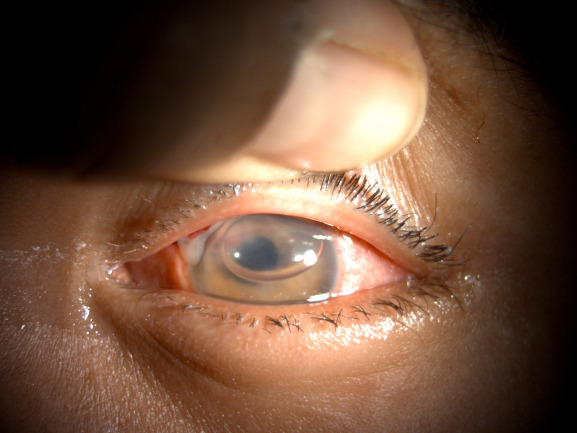
Post-operative Day 1 photograph of the same child (**Fig. 5**). The patient progressed to achieved a visual acuity of 20/ 20 6 months after treatment

**Fig. 7:**
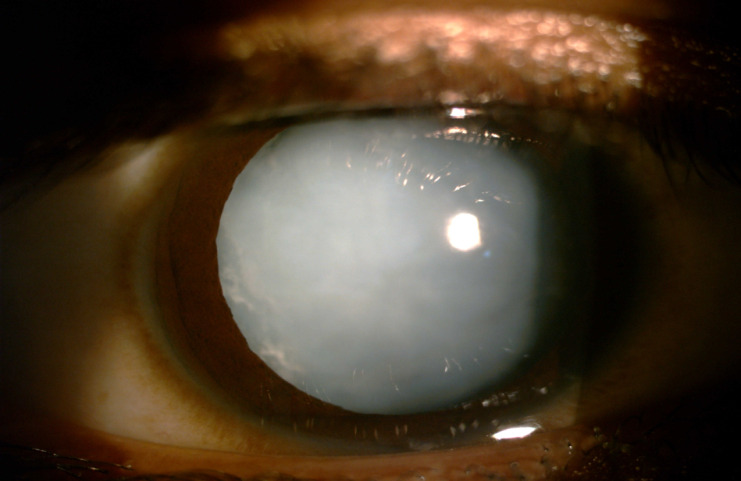
Pre-operative photograph of an 8-year-old male with traumatic cataract who presented to us more than 3 months after blunt trauma

**Fig. 8:**
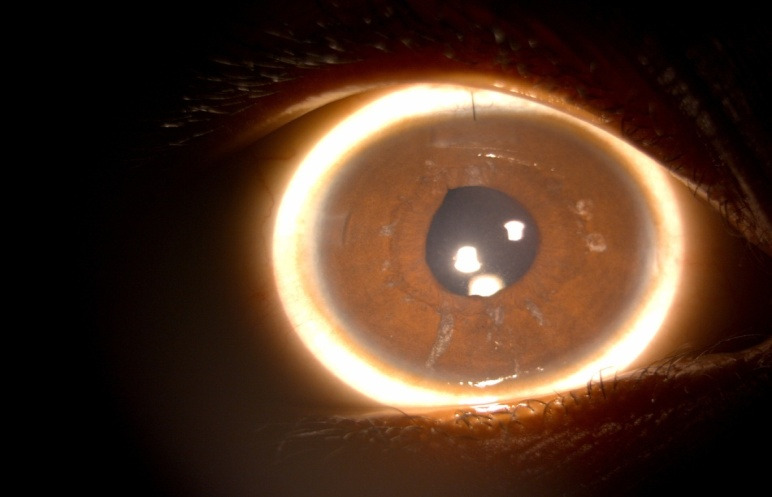
Post-operative Day 1 photograph of the same child (**Fig. 7**). The patient went on to achieve a visual acuity of 20/ 32, 6 months after surgery

**Fig. 5** and **6** show the pre-op and post-op pictures, respectively of a 3-year-old male with limbal tear due to penetrating injury. **Fig. 7** and **8** show the pre-op and post-op slit lamp photographs, respectively of an 8-year-old male with blunt trauma leading to traumatic cataract with late presentation. The patient went on to achieve a vision of 20/ 32 following surgery. 

## Discussion

Traumatic ocular injuries often cause significant visual loss in young patients, as well as adults, and is a leading cause of non-congenital, monocular blindness, especially in children. However, visual prognosis in ocular trauma patients can be significantly improved with timely presentation, prompt treatment, regular follow-up, amblyopia treatment, and refraction.

In our study, a higher number of patients were males(n=96,80%). In both OTS and POTS categories, most of the presenting patients were males. Similar findings were reported in a study conducted by Agrawal R et al. [**5**] in 2013. Hong TZ et al. [**6**] found male preponderance in ocular trauma, with 74% cases <40 years of age. Martina M et al. [**7**] also confirmed that most patients with ocular trauma were boys, on a male: female ratio close to 7:3, though not statistically significant to the final visual outcome. Considering our sample population, North India is a largely patriarchic state, with a male majority dominating all aspects of outdoor work, while the female population is generally concerned with the household chores, thereby predisposing the male population to more trauma, ocular or otherwise.

All the injuries were unilateral, but there was a higher preponderance of left eye injury (n=74, 61.67%), than right eye injury. This might be attributed to dexterity, as people tend to protect their right eye better than the left one. However, no other studies have mentioned any such associations among them. In our study, laterality of the involved eye was not relevant to the final visual outcome in any way.

The patients, both males and females, were mostly young, presumably working adults, below the age of 40 years. In this study, 91.6% patients included were under the age of 40 years. Only 10 out of 120 patients (8.33%) were >40 years age. In International Globe and Adnexal Trauma Epidemiology Study (IGATES), Hong TZ et al. [**6**] confirmed these findings, as 81% of their patients were below the age of 40 years. These demographic findings reflect the involvement of younger, more aggressive males in higher risk activities [**6**]. Both Krishnaiah et al. [**8**] and Nirmalan et al. [**9**] support these findings by pointing out that males are more likely to suffer from open globe injuries, with occupational hazard being the most common cause.

As observed in our study, blunt ocular trauma was more common than penetrating ocular trauma, the former being 51.66% of all cases. Several studies have been conducted in different parts of the world regarding the epidemiology of ocular trauma. In their 2007 study of urban slums in North India, Vats et al. [**10**] included a sample very similar to our study, and observed that blunt ocular trauma was the most common mode of ocular trauma, with 41.7% injuries attributed to blunt trauma. However, the Beaver Dam Study [**11**] performed in United States of America (USA), in 2000, observed that the most common cause of ocular trauma was from sharp objects (54.1%), resulting in penetrating injuries.

Our study found OTS to be significantly predictive (p value=0.002) of final visual acuity post-operatively. 62.5% (n=10) of those having maximum probability to achieve a VA of PL+/ HM, achieved the same. 57.14% (n=32) of the patients with maximum probability to have a final VA of 20/ 40 or better, achieved the same. The low percentages might be attributed to the unequitable distribution in the probability table, whereby the maximum probability of final VA was either in Category 1 or Category 5. That is, either the vision achieved was very good or very poor. Previous studies, like the one of Lesniac et al. [**12**], have reported no significant difference between final visual acuities and the visual acuities predicted by OTS in children. Shah et al. [**13**] also observed a good correlation of OTS with the final visual outcome, and they recommended that OTS may be a reliable tool to predict the visual outcome in pediatric trauma cases. Unver et al. [**14**] found that OTS may provide only a gross prediction of the final visual acuity in pediatric patients with open globe injuries and therefore have limited value in assessing children with traumatic ocular injuries.

POTS was used for pediatric patients with penetrating ocular trauma. In our study, the POTS was observed to provide poor predictions for final VA. 26 patients were predicted to have the maximal probability to achieve the final VA of NPL, out of whom only 2 (7.6%) achieved the final vision. Similarly, none of the patients were predicted to achieve the final VA of 20/ 40 or better, though 18 patients achieved the same. This underestimation of final VA may be attributed to assigning lower scores to initial VA and significant deductions for traumatic cataract, vitreous haemorrhage, Hyphaema and iris prolapse, which have not shown to indicate poor visual prognosis. This finding is opposed to the results of Morgan et al. [**15**], who concluded in their study that both OTS and POTS predicted similar results and both underestimated the potential best corrected visual acuity after treatment. In their study, Awidi et al. [**16**] concluded that POTS correlated better with the final VA in the pediatric population than OTS. However, our results were in coherence with a recent study by Pahor D et al. [**17**], which concluded that OTS is a better predictor of visual prognosis in ocular trauma patients while POTS grossly underestimated the visual potential.

## Conclusion

In this study, OTS was a reliable predictor of final visual outcome, both in blunt and penetrating injuries, in patients of all age groups. OTS delivered consistent results with over a variety of injuries and pathologies. However, even though it has been labelled a reliable tool to estimate the visual prognosis post-trauma in several previously conducted studies, the POTS has not lived up to the expected standard in this study. It showed gross underestimation of final visual outcome in our study. 

Limitations of this study included the small sample size, small follow-up period, and difficulty in actual quantification of presenting visual acuity in preverbal children. This study also lacked the inclusion of traumatic Endophthalmitis and NPL patients, who might have been an addition to poor visual prognosis.

**Conflict of Interest Statement**

The authors state no conflict of interest. 

**Informed Consent and Human and Animal Rights statement**

Informed consent has been obtained from the legal guardians of the patients included in the study.

**Authorization for the use of human subjects**

Ethical approval: The research related to human use complies with all the relevant national regulations, institutional policies, it is in accordance with the tenets of the Helsinki Declaration and has been approved by the review board of Motilal Nehru Medical College, Prayagraj, U.P., India.

**Acknowledgements**

None. 

**Sources of Funding**

None. 

**Disclosures**

None. 
